# Polyporusterone B Alleviates Inflammatory Injury via Suppression of Pro-Inflammatory Cytokine Production

**DOI:** 10.3390/ijms26209957

**Published:** 2025-10-13

**Authors:** Dan Song, Yanru Zhang, Jialu Yuan, Xiaohua Hao, Shizhuo Chen, Xinjie Zhao, Yaomeng Yang

**Affiliations:** 1 Joint Laboratory for Research on Active Components and Pharmacological Mechanism of Tibetan Materia Medica of Tibetan Medical Research Center of Tibet, School of Medicine, Xizang Minzu University, Xianyang 712082, China; 18646783495@139.com (Y.Z.); 15047356350@139.com (J.Y.); 18335429745@139.com (X.H.); 19712525691@163.com (S.C.); 19723032309@139.com (X.Z.); 18935319496@139.com (Y.Y.); 2 Key Laboratory for High Altitude Brain Injury and Repair, School of Medicine, Xizang Minzu University, Xianyang 712082, China

**Keywords:** Polyporusterone B, anti-inflammation, MAPK pathway, NF-κB, endotoxin shock

## Abstract

Polyporusterone B, a triterpene carboxylic acid isolated from *Polyporus umbellatus* Fries, exhibits anti-cancer and anti-hemolytic activities; however, its anti-inflammatory properties and underlying mechanisms remain unelucidated. We studied the anti-inflammatory effects of Polyporusterone B using lipopolysaccharide (LPS)-stimulated Raw264.7 murine macrophages (in vitro) and LPS-induced endotoxin shock in C57BL/6 mice (in vivo). Results showed that Polyporusterone B (1, 5, and 10 μM) had no cytotoxicity toward Raw264.7 cells, but significantly inhibited LPS-induced production of nitric oxide (NO) and pro-inflammatory cytokines (tumor necrosis factor (TNF-α), interleukin 1β (IL-1β), and interleukin 6 (IL-6)) in a concentration- and time-dependent manner, as demonstrated by Griess assay, qPCR, and ELISA. Western blot analysis revealed that Polyporusterone B suppressed LPS-induced phosphorylation of mitogen-activated protein kinases (ERK, P38, and NK) and reduced phosphorylation-mediated degradation of inhibitor of κBα (IκBα). Immunofluorescence and immunohistochemical staining further confirmed that Polyporusterone B blocked nuclear translocation of nuclear factor kappa-B (NF-κB)/Rel A in both Raw264.7 cells and mouse tissues. In the in vivo model, Polyporusterone B pretreatment significantly mitigated LPS-induced multi-organ pathological damage (e.g., lung edema, hepatic inflammation, renal hemorrhage) and downregulated tissue levels of TNF-α, IL-1β, and IL-6. These findings suggest that Polyporusterone B exerts anti-inflammatory effects by inhibiting the mitogen-activated protein kinase (MAPK) and NF-κB signaling pathways, suggesting its potential as a therapeutic candidate for inflammatory diseases.

## 1. Introduction

Inflammation is a conserved innate immune response triggered by pathogens, tissue damage, or other harmful stimuli, serving to eliminate insults and restore tissue homeostasis [1]. Macrophages are central regulators of inflammation: upon activation, they secrete a cascade of pro-inflammatory mediators and activate signaling pathways that amplify immune responses [2,3]. Toll-like receptors (TLRs) are conserved molecular sensors that identify pathogen-associated molecular patterns [4]. Lipopolysaccharide (LPS) is a component of the outer membrane of Gram-negative bacteria that is recognized by TLR4, resulting in activation of myeloid differentiation factor 88 (MyD88) and the toll–interleukin-1 receptor (TIR) domain-containing adapter [5]. Activated interleukin receptor-associated kinase-1 (IRAK) family members are isolated from MyD88 and interact with tumor necrosis factor (TNF) receptor-associated factor 6 (TRAF6) [6]. Subsequently, IRAK-1 phosphorylates TRAF6 and activates the TGF-β-activated kinase 1 (TAK1)–TAK1-binding protein (TAB) complex [7]. TAK1 activates the trimeric IκB kinase (IKK) complex by phosphorylation of IKKβ, then phosphorylates IκBα, mediates its degradation, promotes the translocation of activated NF-κB to the nucleus, and initiates the transcription of inflammation-related genes such as interleukin 1β (IL-1β), TNF-α, and interleukin 6 (IL-6) [8,9]. TAK1 activation has also been shown to be related to the activation of the MAPKs, which are composed of a three-tier kinase cascade, including extracellular signal-regulated kinase (Erk), P38, and c-Jun N-terminal kinase (JNK), that results in the dual-phosphorylation and activation of MAPKs, which trigger activator protein-1 and ultimately initiate the transcription of inflammation-related genes [10].

The activation of the NF-κB and MAPK signaling pathways leads to excessive innate immune response, leading to acute and chronic inflammatory diseases, such as neurodegenerative disorders [11], inflammatory bowel disease (IBD) [12], chronic obstructive pulmonary disease (COPD), rheumatoid arthritis (RA), and cancer [13]. TLRs are regulators of the inflammatory immune response. The negative regulation of the TLR signal is necessary for avoiding an excessive inflammatory immune response and maintaining immune homeostasis [14,15].

Natural products are a rich source of bioactive compounds with anti-inflammatory potential. Polyporusterone B is a triterpene carboxylic acid isolated from *Polyporus umbellatus* Fries that inhibits free radical-induced red blood cell lysis [16]. Polyporusterone B and other compounds isolated from *Polyporus umbellatus* showed significant anti-cancer activity [17]. Polyporusterone B displays an anti-cancer effect; however, it has not been found to have an anti-inflammatory effect.

In this study, we aimed to investigate the anti-inflammatory potential of Polyporusterone B using LPS-stimulated Raw264.7 macrophages and LPS-induced endotoxin shock in mice. We further explored whether its effects are mediated by regulating the NF-κB and MAPK signaling pathways, providing a scientific basis for Polyporusterone B’s development as an anti-inflammatory agent.

## 2. Results

### 2.1. Polyporusterone B Inhibits LPS-Induced NO Production

**Figure 1:**
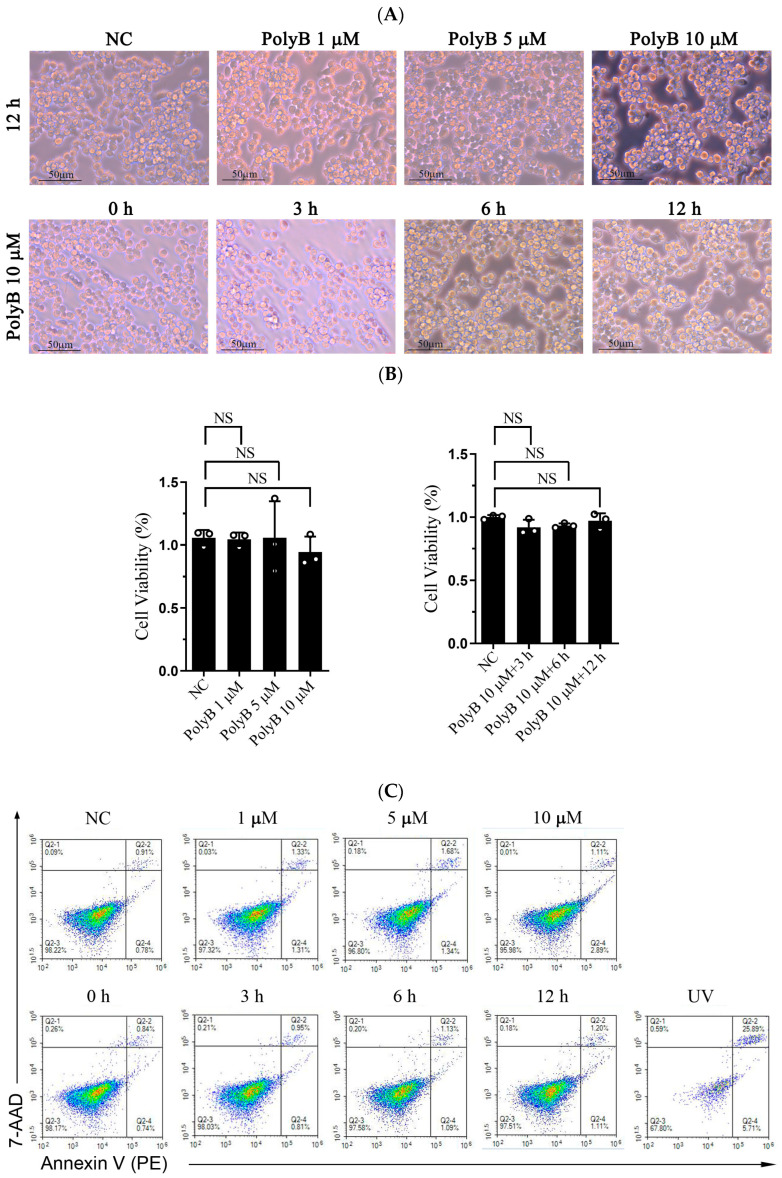

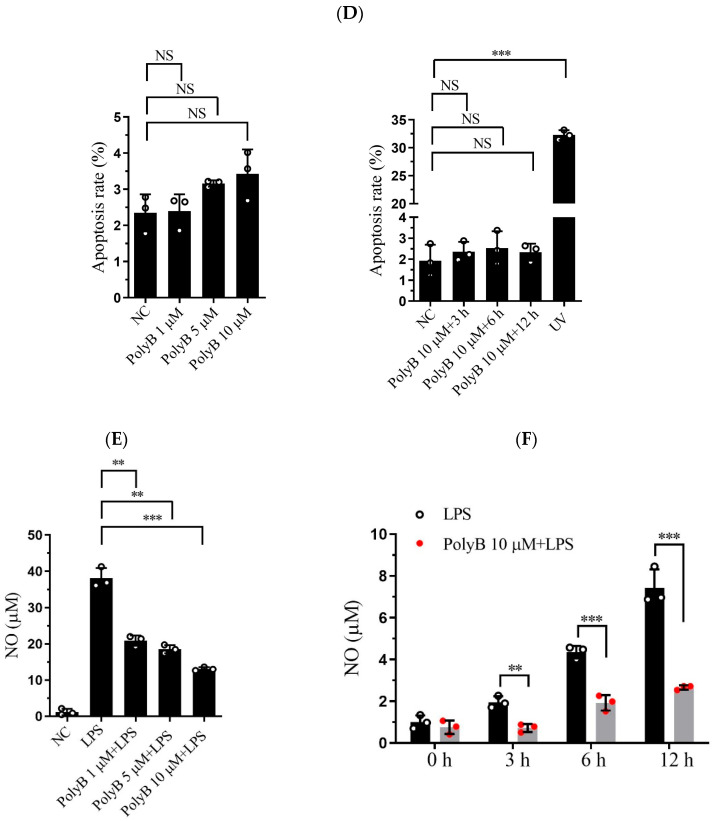
Polyporusterone B inhibits LPS-induced NO production. The Raw264.7 cells were pre-treated with Polyporusterone B followed by LPS stimulation for different time points (0, 3, 6, or 12 h) or with different concentrations (0, 1, 5, or 10 μM) of Polyporusterone B followed by LPS stimulation for 12 h. The supernatants were collected and assayed using the Griess method. (**A**,**B**) The morphological observation of Polyporusterone B-treated cells. (**C**,**D**) The flow cytometry results of Polyporusterone B-treated cells. (**E**,**F**) The expression level of NO. *n* = 3. NS, not significant. ** *p* < 0.01, and *** *p* < 0.001.

Polyporusterone B is a triterpene carboxylic acid isolated from *Polyporus umbellatus* Fries. To rule out potential cytotoxic effects of Polyporusterone B on Raw264.7 cells, we performed morphological observation and flow cytometry analysis (Figure 1A,B). Neither assay detected signs of cell apoptosis induced by Polyporusterone B, confirming that Polyporusterone B exhibits no cytotoxicity toward Raw264.7 cells under the experimental conditions used. NO, a key pro-inflammatory mediator, serves as a critical indicator of inflammatory responses during innate immunity [18]. Given its role in LPS-induced inflammation, we quantified NO production by measuring nitrite accumulation (a stable metabolite of NO) using the Griess reagent assay. As expected, stimulation of Raw264.7 cells with LPS resulted in a significant increase in nitrite concentration compared to untreated controls. Notably, this LPS-induced nitrite elevation was markedly attenuated when cells were pretreated with Polyporusterone B at concentrations of 1, 5, or 10 μM (Figure 1C). To further characterize the inhibitory effect of Polyporusterone B, we assessed its time-dependent impact on LPS-induced NO production. Pretreatment with 10 μM Polyporusterone B for 3, 6, or 12 h consistently suppressed NO generation in LPS-stimulated Raw264.7 cells (Figure 1D).

Collectively, these findings demonstrate that Polyporusterone B inhibits LPS-induced NO production in Raw264.7 cells in both a concentration- and time-dependent manner.

### 2.2. Polyporusterone B Inhibits Pro-Inflammatory Cytokines TNF-α and IL-6 Expression

**Figure 2:**
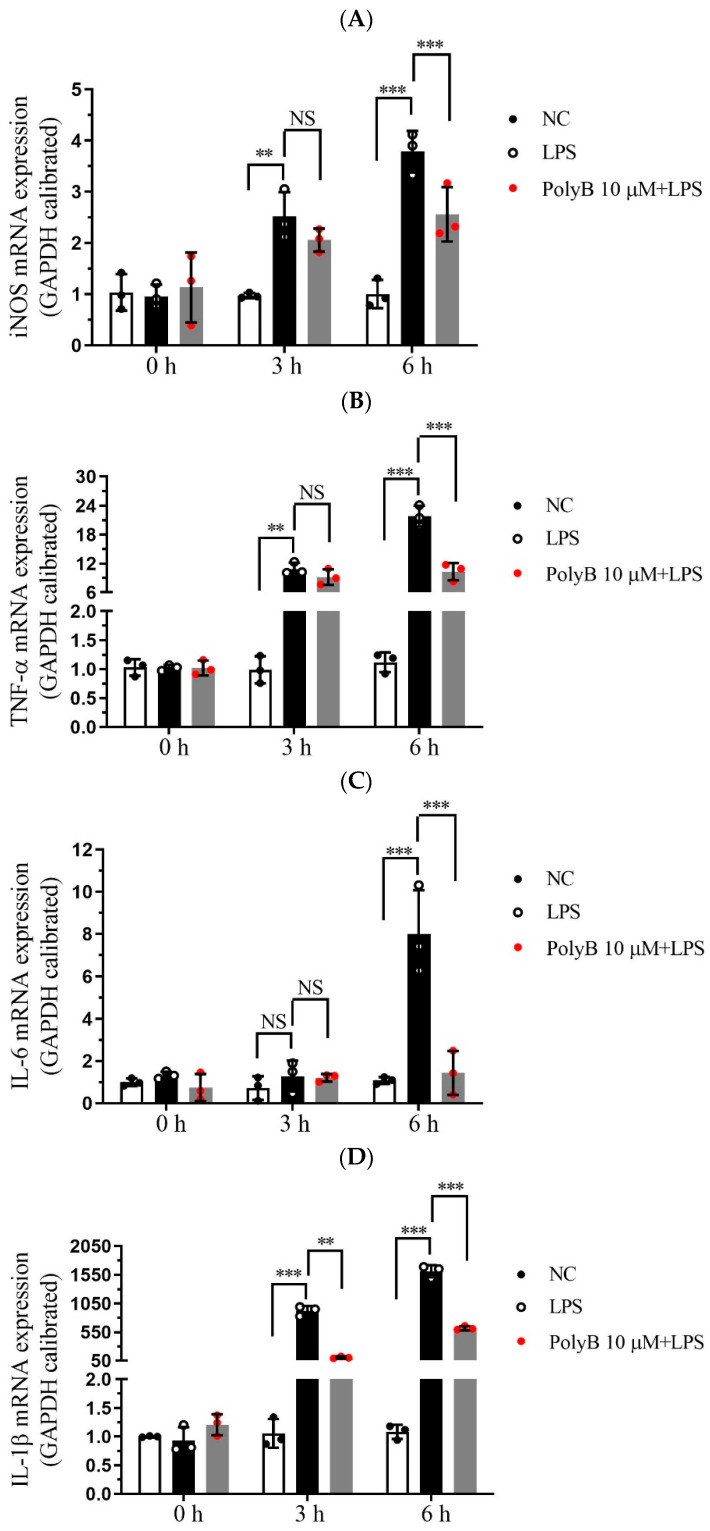

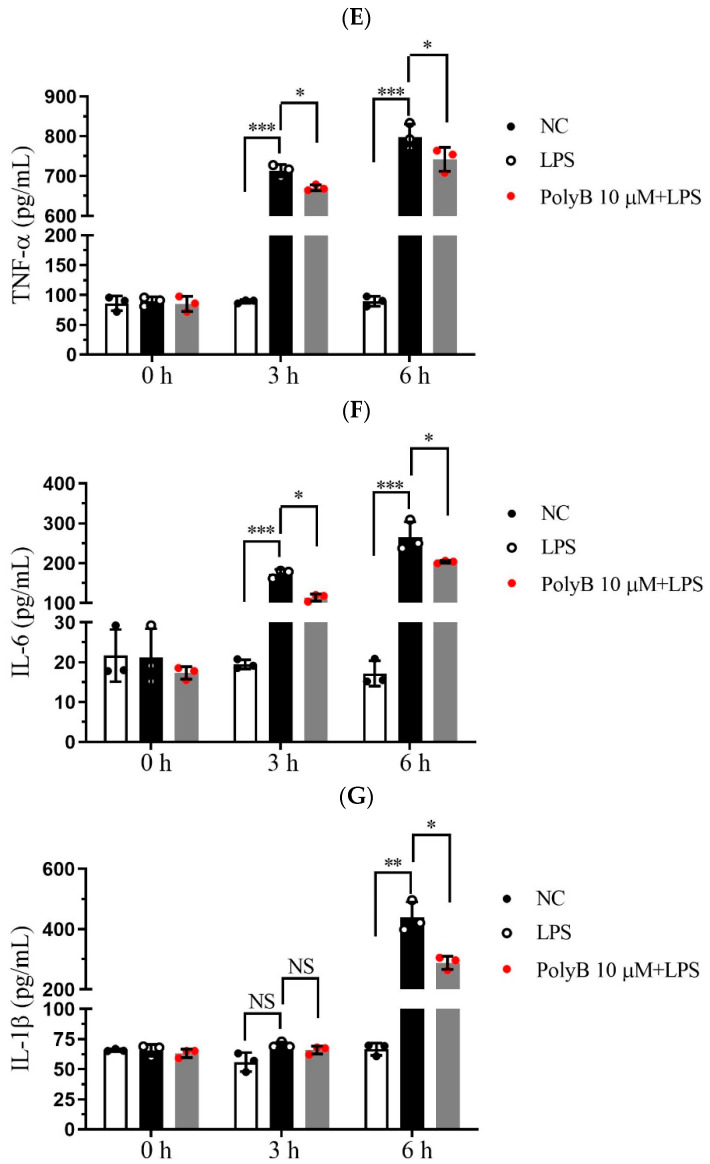
Polyporusterone B inhibits pro-inflammatory cytokine expression. Raw264.7 cells were pretreated with 10 μM Polyporusterone B for 3 h, then treated with LPS at different time points (0, 3, or 6 h). The mRNA levels of (**A**) iNOS, (**B**) TNF-α, (**C**) IL-6, and (**D**) IL-1β were measured by qPCR. The cytokine levels of (**E**) TNF-α, (**F**) IL-6, and (**G**) IL-1β were tested by ELISA. *n* = 3. NS, not significant. * *p* < 0.05, ** *p* < 0.01, and *** *p* < 0.001.

Pro-inflammatory cytokines, including TNF-α, IL-6, and IL-1β, are pivotal effector molecules secreted by macrophages that drive the progression of inflammatory responses. Given that inducible nitric oxide synthase (iNOS) is a key enzyme mediating NO production and closely linked to inflammatory processes, we extended our investigations to include iNOS alongside the aforementioned cytokines. Building on our previous findings that Polyporusterone B inhibits LPS-induced NO production, we next investigated whether Polyporusterone B could modulate the expression and secretion of these key pro-inflammatory cytokines as well as iNOS in Raw264.7 cells. To this end, we employed qPCR to assess the transcriptional levels of iNOS, TNF-α, IL-6, and IL-1β, and ELISA to quantify their secretion into the cell culture supernatants. Consistent with our earlier observations of LPS-induced NO elevation, qPCR analysis revealed that LPS stimulation led to a significant upregulation of iNOS mRNA expression. Importantly, pretreatment with Polyporusterone B markedly suppressed this LPS-induced increase in iNOS transcription, which aligns with its inhibitory effect on NO production (Figure 2A). Figure 2B–D showed that at the transcriptional level, pretreatment with Polyporusterone B significantly downregulated the LPS-induced upregulation of TNF-α, IL-6, and IL-1β mRNA expression. At the protein secretion level, ELISA results revealed that Polyporusterone B pretreatment markedly reduced the secretion of TNF-α and IL-6 triggered by LPS stimulation; however, while there was a detectable inhibitory trend on IL-1β secretion, this effect did not reach statistical significance (Figure 2E–G).

These results indicate that Polyporusterone B exerts a suppressive effect on LPS-induced pro-inflammatory cytokine production in Raw264.7 cells, showing consistent inhibitory effects on the pro-inflammatory cytokines TNF-α and IL-6 at both the transcriptional and translational levels, and also significantly downregulates the transcriptional level of iNOS, thus aligning with our earlier observation that Polyporusterone B inhibits LPS-induced NO generation. For IL-1β, the significant downregulation at the mRNA level but lack of statistical significance at the protein secretion level may reflect potential post-transcriptional regulatory mechanisms that modulate IL-1β’s final secretion or other related processes. This discrepancy warrants further investigation to clarify the precise regulatory pattern of Polyporusterone B on IL-1β.

### 2.3. Polyporusterone B Inhibits LPS-Induced Phosphorylation of the MAPK Signaling Pathway and the Nuclear Localization of NF-κB

**Figure 3:**
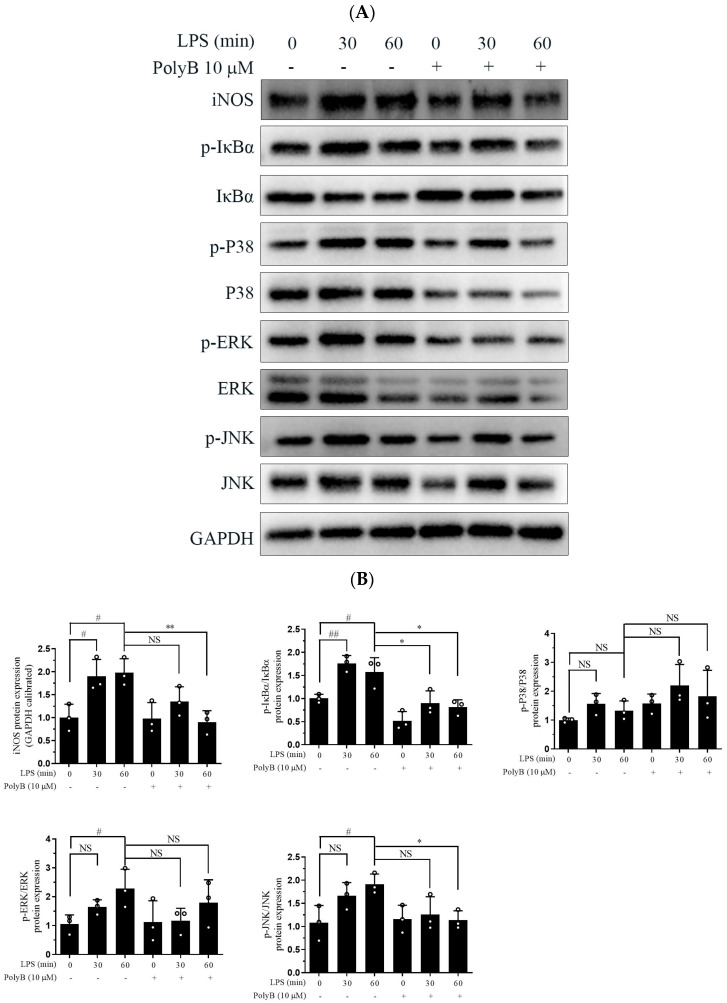

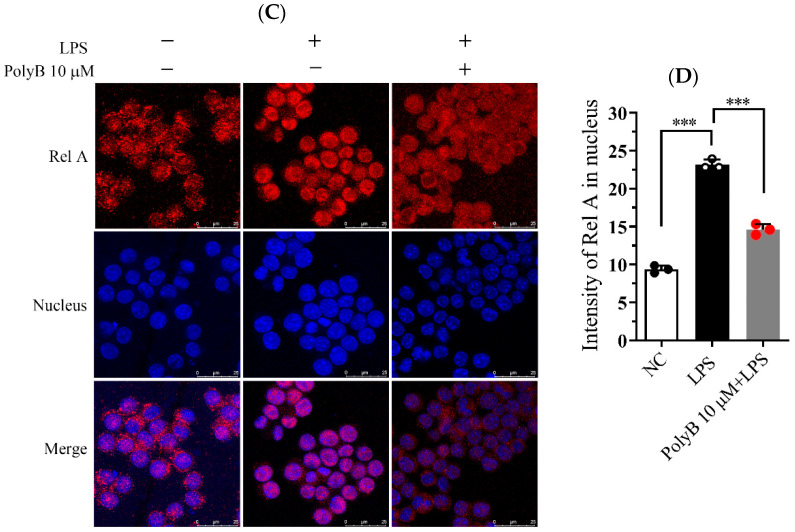
Polyporusterone B inhibits LPS-induced phosphorylation of the MAPK signaling pathway and the nuclear localization of NF-κB. (**A**) Raw264.7 cells were pretreated with 10 μM Polyporusterone B for 3 h, then stimulated with LPS for 0, 30, or 60 min. Cells were lysed and assayed for GAPDH, p-ERK, p-P38, p-JNK, IκBα, and p-IκBα by Western blots. (**B**) Quantitative analysis of WB. *n* = 3. # *p* < 0.05 and ## *p* < 0.01 vs. LPS 0 min group. * *p* < 0.05 and ** *p* < 0.01 vs. LPS 60 min group. (**C**) Raw264.7 cells were pretreated with 10 μM Polyporusterone B for 3 h, followed by stimulation with LPS for 30 min. Immunofluorescence staining was observed by Leica laser scanning spectral confocal microscope; the scale bar is 25 μm. (**D**) Quantitative analysis of immunofluorescence staining. *n* = 3. *** *p* < 0.001.

The MAPK signaling pathway, which includes P38, Erk, and JNK, is tightly intertwined with the initiation and amplification of inflammatory responses [19]. To dissect the underlying molecular mechanisms of Polyporusterone B’s anti-inflammatory activity, we first examined the phosphorylation status of key MAPK family members using Western blot analysis. Western blot results showed that Polyporusterone B pretreatment inhibited the LPS-induced phosphorylation of P38, ERK, and JNK (Figure 3A). NF-κB is a critical transcription factor that regulates various inflammation-related genes [20]. The inhibitor of κBα (IκBα) acts as a crucial negative regulator of NF-κB activation: in the resting state, IκBα sequesters NF-κB in the cytoplasm, and LPS-induced phosphorylation of IκBα triggers its ubiquitination and degradation, thereby releasing NF-κB for nuclear translocation. Consistent with this, our Western blot results showed that LPS treatment significantly increased IκBα phosphorylation and reduced total IκBα protein levels (indicative of IκBα degradation). Significantly, Polyporusterone B pretreatment reversed the LPS-induced decrease in total IκBα, suggesting that it inhibits LPS-mediated IκBα degradation (Figure 3A,B). These findings imply that Polyporusterone B may exert its anti-inflammatory effects, at least in part, by modulating the NF-κB signaling pathway.

Since nuclear translocation is a prerequisite for NF-κB activation and subsequent transcriptional regulation of target genes, we further evaluated the impact of Polyporusterone B on NF-κB/Rel A nuclear translocation using immunofluorescence staining. As depicted in Figure 3C,D, LPS stimulation led to a pronounced accumulation of NF-κB/Rel A in the nucleus of Raw264.7 cells. In contrast, pretreatment with Polyporusterone B clearly suppressed the LPS-induced nuclear translocation of NF-κB/Rel A.

Taken together, these results demonstrate that Polyporusterone B inhibits LPS-induced inflammatory responses by suppressing both the phosphorylation of the MAPK signaling pathway and the activation of the NF-κB pathway.

### 2.4. Polyporusterone B Ameliorates Organ Injuries in LPS-Induced Endotoxin Shock

**Figure 4:**
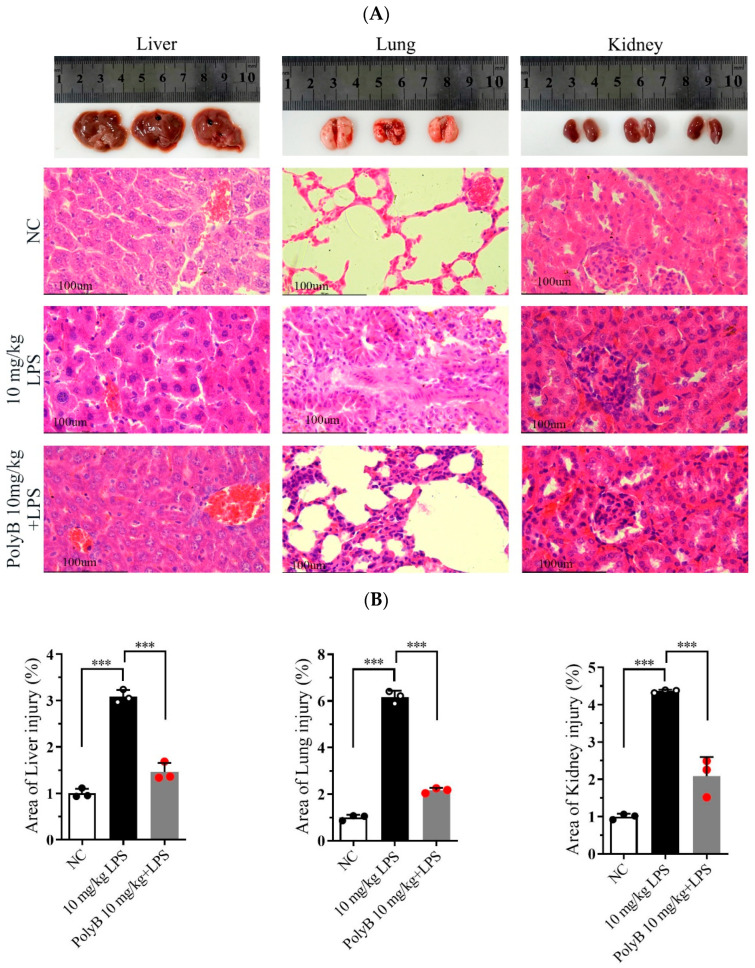

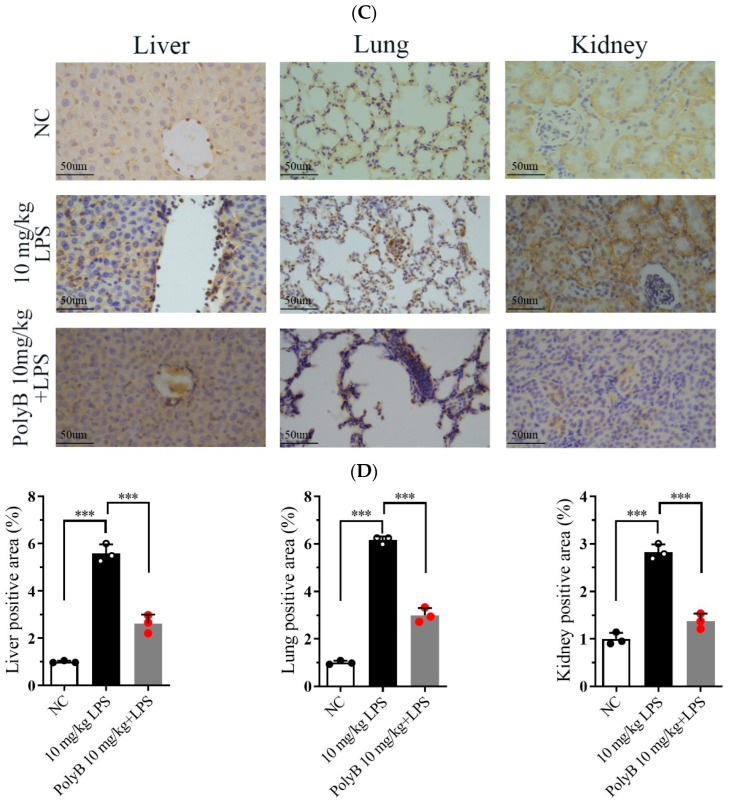
Polyporusterone B ameliorates organ injuries in LPS-induced endotoxin shock. Images of organs in different groups: representative morphological images by (**A**) H&E staining (the scale bar is 100 μm) and (**B**) quantitative analysis of H&E; (**C**) IHCstaining (the scale bar is 50 μm) and (**D**) quantitative analysis of IHC were presented to assess lung, liver, and kidney injury. *n* = 3. *** *p* < 0.001.

Hematoxylin and Eosin (H&E) staining was performed to evaluate tissue structural integrity and pathological changes, while IHC analysis was used to characterize further protein expression associated with inflammatory responses. As shown in Figure 4A,B, compared with the normal control group, mice subjected to LPS-induced endotoxin shock exhibited severe pathological damage in multiple organs. Specifically, lung tissue displayed marked interstitial edema, focal hemorrhage, significant thickening of alveolar walls, massive infiltration of inflammatory cells, and disruption of alveolar structure with partial alveolar collapse. Liver tissue displayed diffuse infiltration of inflammatory cells around central veins and portal areas, accompanied by mild hepatocyte swelling. Kidney tissue showed prominent interstitial inflammatory cell infiltration, scattered punctate hemorrhage in the renal cortex, and mild tubular epithelial cell degeneration. Notably, pretreatment with Polyporusterone B significantly alleviated these LPS-induced pathological changes: lung alveolar structure was partially preserved, with reduced edema and inflammatory infiltration; hepatic inflammatory cell accumulation was diminished; and renal interstitial inflammation and hemorrhage were visibly mitigated. Consistent with the H&E results, Immunohistochemistry (IHC) analysis in Figure 4C,D showed that LPS-induced nuclear translocation of NF-κB/Rel A in lung, liver, and kidney tissues was significantly inhibited by Polyporusterone B pretreatment, further confirming the suppression of local inflammatory responses.

These histological and immunohistochemical findings collectively demonstrate that Polyporusterone B effectively mitigates LPS-induced multi-organ pathological damage in endotoxin shock, which is closely associated with its inhibition of local tissue inflammatory responses.

### 2.5. Polyporusterone B Modulates Inflammatory Cytokine Levels and Improves Organ Function In Vivo

Building on our in vitro findings that Polyporusterone B pretreatment downregulates LPS-induced increases in TNF-α, IL-1β, and IL-6 in Raw264.7 cells, we next validated its in vivo anti-inflammatory effects using an LPS-induced endotoxin shock mouse model. We assessed the transcriptional and protein levels of key inflammatory mediators in target tissues, including the lung, liver, and kidney. We analyzed serum cytokines and organ function biomarkers to evaluate their efficacy comprehensively.

**Figure 5:**
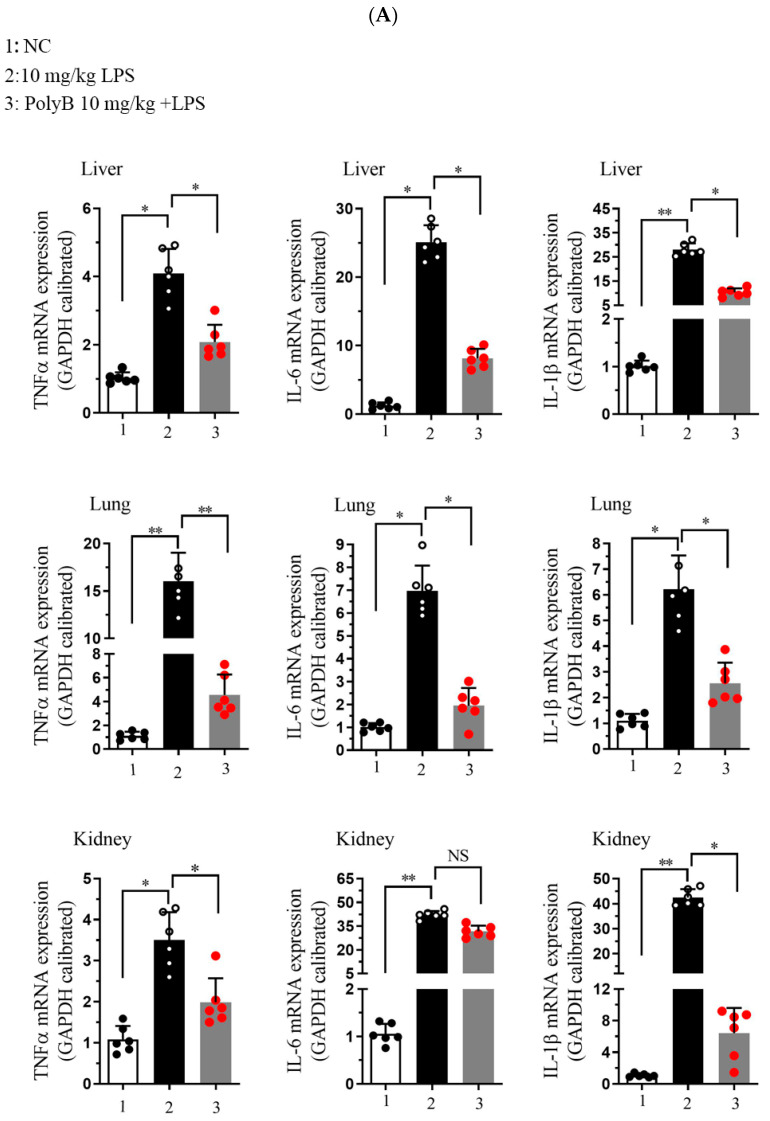

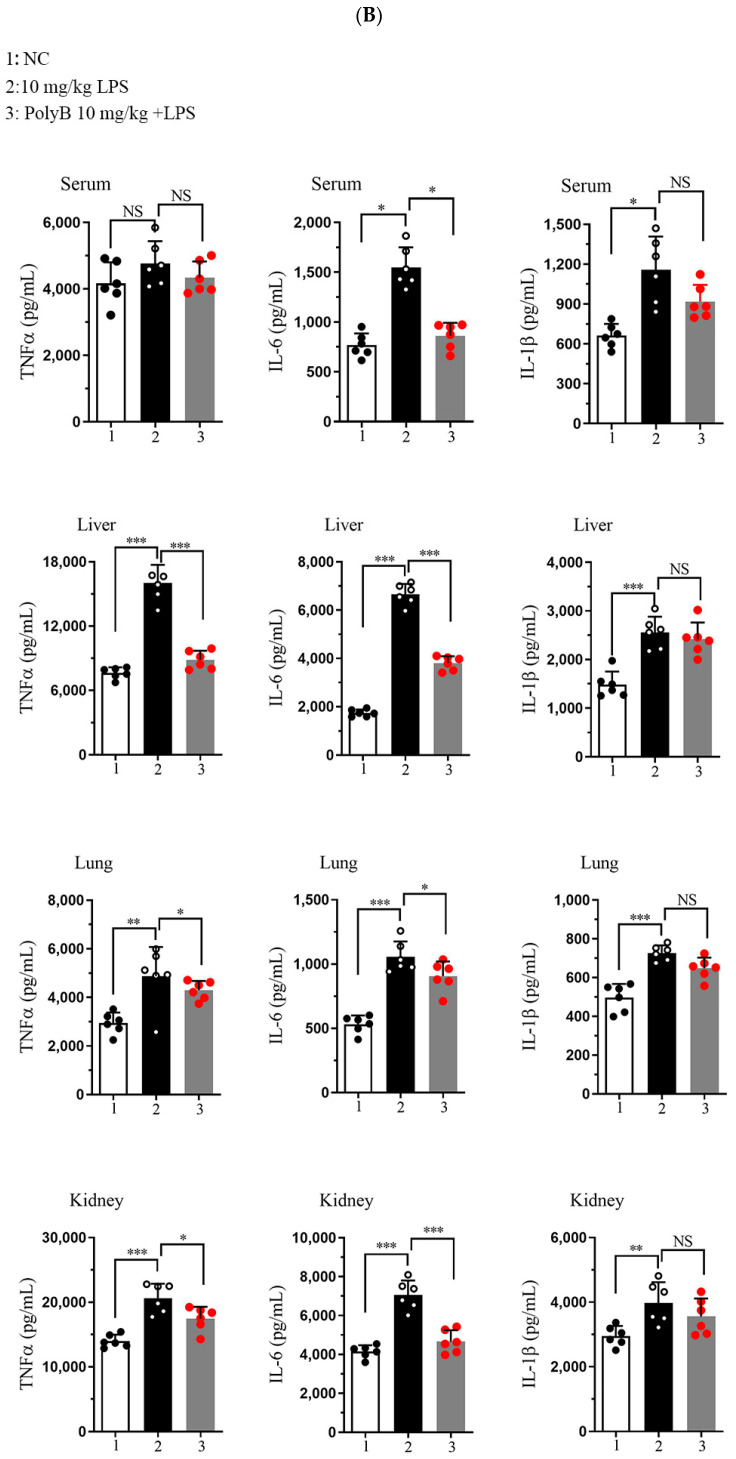

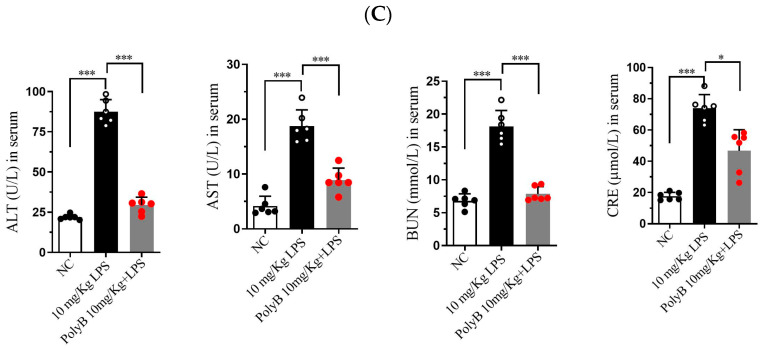
Polyporusterone B reduces the concentration of inflammatory cytokines in vivo. (**A**) The mRNA expression of TNF-α, IL-1β, and IL-6 was analyzed by qPCR. (**B**) ELISA analyzed the release of TNF-α, IL-1β, and IL-6 cytokines. (**C**) Serum biomarkers of ALT, AST, BUN, and CRE. *n* = 6. NS, not significant. * *p* < 0.05, ** *p* < 0.01, and *** *p* < 0.001.

At the transcriptional level, as shown in Figure 5A, LPS challenge significantly upregulated the mRNA expression of TNF-α, IL-1β, and IL-6 in all three tissues. Pretreatment with Polyporusterone B significantly suppressed these LPS-induced elevations for all three cytokines. The serum cytokine analyses in Figure 5B show that Polyporusterone B significantly inhibited LPS-induced IL-6 protein elevation, while TNF-α and IL-1β levels trended downward but did not reach statistical significance. Meanwhile, tissue-specific differences emerged at the protein level: Polyporusterone B significantly reduced LPS-induced TNF-α protein levels in the lung, liver, and kidney, but its effects on IL-1β and IL-6 were more variable. Specifically, in lung tissue, IL-1β protein levels showed a downward trend, but this change did not reach statistical significance after Polyporusterone B treatment; in liver and kidney, IL-1β protein levels similarly trended lower without reaching significance. This pattern aligns with our in vitro observations, where IL-1β secretion also showed a non-significant reduction despite transcriptional suppression, suggesting potential post-transcriptional regulatory mechanisms that warrant further exploration. To further link cytokine modulation to functional outcomes, we identified key serum biomarkers; as shown in Figure 5C, LPS challenge significantly increased serum levels of AST and ALT, the indicators of hepatic dysfunction, as well as BUN and CRE, the indicators of renal dysfunction. Importantly, Polyporusterone B pretreatment significantly reduced these LPS-induced elevations, indicating improved hepatic and renal function.

These in vivo results confirm that Polyporusterone B exerts anti-inflammatory effects by suppressing the transcription of pro-inflammatory cytokines across tissues, with variable protein-level regulation that may reflect context-dependent post-transcriptional mechanisms. Its ability to ameliorate LPS-induced organ dysfunction, as evident from improved serum biomarkers, further supports its potential as a multi-organ protective agent in inflammatory conditions.

## 3. Discussion

Our study reports the anti-inflammatory activity of Polyporusterone B and its underlying molecular mechanisms. We demonstrated that Polyporusterone B inhibits LPS-induced inflammation both in vitro (Raw264.7 macrophages) and in vivo (LPS-induced endotoxin shock mice) by suppressing the MAPK and NF-κB signaling pathways, without exhibiting cytotoxicity. These findings expand the known bioactivities of Polyporusterone B, beyond its previously reported anti-cancer and anti-hemolytic effects [16,17], and highlight its potential as a natural anti-inflammatory therapeutic.

The anti-inflammatory effects and molecular mechanisms of Polyporusterone B have not been reported. Our findings suggest that Polyporusterone B inhibits NO synthesis and the expression of LPS-induced pro-inflammatory genes. We demonstrated for the first time the mechanisms by which Polyporusterone B exerts its anti-inflammatory effect and found that it significantly inhibits the NF-κB pathways involved in inflammation regulation.

Inflammation is the primary response of the host to harmful stimuli such as infectious microorganisms or pathogens, and necrosis [21]. At the inflammation site, neutrophils are recruited early, and macrophages are recruited in the late stage [22]. Macrophages are critical regulators that balance the host’s inflammatory response [23]. A recent study demonstrated that LPS can significantly increase NO production in macrophages, resulting in physiological dysfunction [24]. Here, we found that, in LPS-stimulated Raw264.7 cells, Polyporusterone B pretreatment significantly reduced NO levels in a dose-dependent manner. We also found that Polyporusterone B pretreatment reduced the LPS-induced production of cytokines.

The MAPK pathway (ERK, P38, and JNK) is a central regulator of macrophage-mediated inflammation, linking TLR4 activation to the transcription of pro-inflammatory genes. Phosphorylation of ERK is associated with cell proliferation and cytokine secretion, while P38 and JNK modulate stress responses and the production of TNF-α and IL-1β [25,26]. Our Western blot results showed that Polyporusterone B significantly inhibited LPS-induced phosphorylation of ERK, P38, and JNK, suggesting that it targets upstream regulators of MAPKs. NF-κB is a critical regulator of inflammatory gene expression, including cytokines, chemokines, and adhesion factors, and plays a vital role in the developing of various inflammatory diseases [27,28]. However, it is critical to acknowledge that direct evidence supporting the suppression of the MAPK and NF-κB pathways by Polyporusterone B is currently limited to in vitro data. In our in vivo experiments, while Polyporusterone B effectively alleviated LPS-induced multi-organ damage and reduced the transcription/protein levels of pro-inflammatory cytokines such as TNF-α and IL-6 in tissues and serum, we did not directly verify changes in MAPK phosphorylation or NF-κB activation in mouse target tissues. Future studies should address this by detecting pathway-specific molecular changes in animal tissues to establish a direct link between pathway suppression and in vivo efficacy.

To determine the effects of Polyporusterone B on the NF-κB pathway, we analyzed the protein degradation of IκBα and the nuclear translocation of NF-κB/Rel A. Western blots showed that Polyporusterone B pretreatment significantly reduced IκBα degradation by inhibiting phosphorylation. When LPS activates NF-κB, it transfers from the cytoplasm to the nucleus [29]. Our immunofluorescence staining results showed that pretreatment with Polyporusterone B inhibited this translocation.

Our findings suggest that Polyporusterone B exhibits anti-inflammatory properties by inhibiting the production of pro-inflammatory cytokines, including NO, TNF-α, IL-1β, and IL-6. In an LPS-induced endotoxin shock mouse model, pretreatment with Polyporusterone B significantly reduced the inflammatory response, suggesting that Polyporusterone B may serve as a treatment for inflammatory-related diseases.

### Limitations

Despite the significant findings, this study has several limitations that should be acknowledged. Firstly, the in vitro experiments were conducted exclusively using the Raw264.7 macrophage cell line. The anti-inflammatory effects of Polyporusterone B in other immune cell types such as neutrophils, dendritic cells, or primary macrophages remain untested, limiting the generalizability of the results to broader cellular contexts. Secondly, MAPK/NF-κB pathway mechanisms rely exclusively on in vitro data; direct evidence for Polyporusterone B’s suppression of the MAPK and NF-κB pathways is derived solely from in vitro analyses in Raw264.7 cells. We did not detect key pathway molecules in LPS-challenged mouse tissues in vivo. This gap prevents definitive confirmation that the same pathway inhibitory mechanisms underpin its in vivo anti-inflammatory effects. Thirdly, the in vivo validation was restricted to an LPS-induced endotoxin shock mouse model. Inflammatory diseases are diverse (e.g., autoimmune disorders and chronic inflammation), and the efficacy of Polyporusterone B in other pathological models requires further investigation to confirm its broader therapeutic potential. Fourthly, an additional limitation of this study is the lack of pharmacokinetic data for Polyporusterone B, which limits our ability to fully link the administered dose to its in vivo bioactive levels. This gap hinders our ability to optimize dosing strategies and fully contextualize the observed protective effects against LPS-induced inflammation. Finally, although we identified the involvement of MAPK and NF-κB pathways, the downstream molecular targets of Polyporusterone B within these pathways have not been explored. A more detailed mechanistic analysis, including protein–protein interactions or post-translational modifications, would enhance the depth of our understanding.

## 4. Materials and Methods

### 4.1. Chemicals and Reagents

Polyporusterone B (Cat# HY-N7693, purity 98.56%) was purchased from MCE (Shanghai, China). LPS (Cat# L2630) was purchased from Sigma Aldrich (St. Louis, MO, USA). Trizol Reagent (Cat# 15596026) was purchased from Thermo Fisher (Waltham, MA, USA). PrimeScript™ RT Reagent Kit with gDNA Eraser (Cat# RR047A) was purchased from Takara Bio (Kusatsu, Shiga, Japan). SYBR^®^ Green-based qPCR kit (Cat# RR820A) was purchased from Takara Bio (Kusatsu, Shiga, Japan). Phospho-ERK antibody (Cat# AP0485), phospho-P38 antibody (Cat# AP0526), phospho-IκBα antibody (Cat# AP0731), and IκBα antibody (Cat# A19714) were purchased from Abclonal (Wuhan, China). JNK antibody (Cat# CPA1947) and GAPDH antibody (Cat# CPA9067) were purchased from Cohesion Biosciences (London, UK). NF-κB Rel A antibody (Cat# TD7003S) was purchased from Abmart (Shanghai, China). DAPI (Cat# P0131) was purchased from Beyotime (Shanghai, China). DMEM medium (Cat# 11965092) was purchased from Thermo Fisher (Waltham, MA, USA). ELISA kits (Mouse TNF-α, Cat# 88-7324-88; Mouse IL-6, Cat# 88-7064-88; and Mouse IL-1β, Cat# 88-7013-77) were purchased from Invitrogen (Waltham, MA, USA).

### 4.2. Cell Culture

The mouse monocyte–macrophage cell line Raw264.7 was cultured in DMEM supplemented with 10% fetal bovine serum (FBS), 100 IU/mL penicillin, and 100 μg/mL streptomycin. Cells were incubated at 37 °C in a humidified atmosphere with 5% CO_2_. Following the corresponding cell treatment, experimental data were collected from three independent cell culture replicates.

### 4.3. NO Analysis

Raw264.7 was subjected to two sets of experimental treatments: In the first set, cells were pretreated with Polyporusterone B at concentrations of 0, 1, 5, or 10 μM for 3 h, followed by stimulation with LPS at 1 μg/mL for 12 h. In the second set, cells were first pretreated with Polyporusterone B at the same concentration gradient (0, 1, 5, or 10 μM) for 3 h, then stimulated with 1 μg/mL LPS. At different time points post-LPS stimulation (0, 3, 6, or 12 h), cells were harvested to analyze the time-course dynamics of Polyporusterone B-mediated regulation on LPS-triggered responses. To evaluate nitric oxide (NO) production, the concentration of nitrite (a stable metabolite of NO) in the culture supernatants was measured using the standard Griess assay. Briefly, a 50 μL aliquot of each sample was mixed with 50 μL of Griess reagent and incubated at 37 °C for 10 min. Subsequently, 50 μL of sulfanilamide solution was added to each reaction mixture, which was further incubated at 37 °C for 10 min to complete the chromogenic reaction. The absorbance of the resulting solution was measured at a wavelength of 540 nm using a microplate reader, and nitrite concentrations were calculated based on a standard curve generated with known concentrations of sodium nitrite.

### 4.4. RNA Extraction and qPCR Analysis

Total RNA was isolated from Raw264.7 cells using Trizol Reagent as per the manufacturer’s protocol; RNA quality was verified via NanoDrop spectrophotometry, and 1 μg of high-quality total RNA was reverse-transcribed into cDNA using a reverse transcription kit under standard conditions (37 °C for 15 min, 85 °C for 5 s). qPCR was performed on a real-time PCR system using a SYBR^®^ Green-based kit. The primers are listed as follows:

TNF-α: F-GAGTGACAAGCCTGTAGCCC; R-GGTGTGGGTGAGGAGCAC.
IL-6: F-CCAAGAGGTGAGTGCTTCCC; R-CTGTTGTTCAGACTCTCTCCCT.
IL-1β: F-GCAACTGTTCCTGAACTCAACT; R-ATCTTTTGGGGTCCGTCAACT.

### 4.5. Enzyme-Linked Immunosorbent Assay (ELISA)

To quantify the concentrations of target inflammatory factors: TNF-α, IL-6, and IL-1β, Raw264.7 cell culture supernatants and mouse serum samples were collected and analyzed using commercial ELISA kits following the manufacturers’ recommended protocols. After completing the ELISA reaction, the absorbance of each well was measured at a wavelength of 450 nm using a microplate reader. The concentration of each inflammatory factor in the samples was calculated by comparing the measured absorbance values to a standard curve generated with known concentrations of the corresponding recombinant inflammatory factor.

### 4.6. Western Blot (WB)

Raw264.7 cell lysates were prepared and separated by 10% sodium dodecyl sulfate–polyacrylamide gel electrophoresis (SDS-PAGE). After electrophoresis, proteins were electrotransferred onto polyvinylidene difluoride (PVDF) membranes at a constant current. The PVDF membranes were then blocked with 5% bovine serum albumin (BSA) at room temperature for 1 h, with gentle shaking. Following blocking, membranes were incubated overnight at 4 °C with primary antibodies specific to the target proteins diluted in blocking solution. After three washes with TBST, membranes were incubated with horseradish peroxidase (HRP)-conjugated secondary antibodies at room temperature for 1 h with gentle shaking. Membranes were subsequently washed three times with TBST again to remove unbound secondary antibodies, prior to signal detection. To ensure reproducibility, all Western blot experiments were repeated at least three times independently. Band density was quantified using ImageJ software (Image-Pro Plus Version 6.0) and normalized against the internal control GAPDH.

### 4.7. Immunofluorescence Staining

Raw264.7 cells were pretreated with 10 μM Polyporusterone B for 3 h, then treated with LPS for 30 min. Cells were fixed with 4% paraformaldehyde for 10 min and permeabilized with 1% Triton X-100 for 10 min, followed by blocking in PBS containing 5% BSA for 30 min. Primary and secondary antibodies were diluted in PBS containing 5% BSA, and the primary antibody was incubated overnight at 4 °C, followed by the secondary antibody for 30 min at room temperature. Fluorescent images were observed using Leica laser scanning spectral confocal microscope (Nussloch, Germany).

### 4.8. Animal Treatment

Specific pathogen-free male C57BL/6 mice (7–10 weeks) were obtained from Xi’an Jiaotong University. C57BL/6 mice were maintained on a 12/12 h light/dark cycle with ad libitum access to food and water. Mice were randomly divided into three groups: (1) control group with PBS; (2) 10 mg/kg bodyweight Polyporusterone B + 10 mg/kg body weight LPS, and (3) 10 mg/kg body weight LPS. Mice were intraperitoneally (ip) injected with Polyporusterone B or PBS for 3 h, then ip injected with LPS for 6 h. Mice were sacrificed for analysis. All procedures were conducted in accordance with the “Guiding Principles in the Care and Use of Animals” (China).

### 4.9. Histological and Immunohistochemical Analysis

After sacrifice, target mouse tissues lung, liver, and kidney were rapidly dissected, rinsed with cold PBS to remove blood contaminants, and immediately fixed in 4% (*v*/*v*) paraformaldehyde for 24 h. Fixed tissues were sequentially dehydrated through a graded ethanol series, cleared in xylene, and embedded in paraffin wax to prepare tissue blocks. Paraffin blocks were sectioned into 5 μm thick slices using a microtome, and dried overnight at 37 °C.

For hematoxylin and eosin (H&E): sections were stained with hematoxylin for nuclear visualization, differentiated with 1% hydrochloric acid–ethanol, counterstained with eosin for cytoplasmic and extracellular matrix visualization, then dehydrated, cleared, and mounted with neutral balsam.

For immunohistochemical (IHC) analysis targeting RelA: deparaffinized and rehydrated sections were subjected to antigen retrieval by boiling in 0.01 M sodium citrate buffer (pH 6.0) for 15 min, followed by cooling at room temperature for 20 min. Sections were blocked with 5% BSA for 1 h at 37 °C to reduce non-specific binding, then incubated overnight at 4 °C with a primary anti-Rel A antibody. After three washes, sections were incubated with a horseradish HRP-conjugated secondary antibody for 1 h at 37 °C. Signal was developed using 3,3′-diaminobenzidine (DAB) chromogen substrate, and sections were counterstained lightly with hematoxylin, then dehydrated, cleared, and mounted.

### 4.10. Statistical Analysis

Experimental data are presented as mean ± standard deviation. Normality was assessed using the Anderson–Darling tests. Post hoc and multiple comparison tests were applied for further analysis. A one-way analysis of variance (ANOVA) was utilized to evaluate the statistical significance of differences among groups.

## 5. Conclusions

Polyporusterone B exerts anti-inflammatory effects both in vitro and in vivo. In vitro, it is non-cytotoxic to Raw264.7 macrophages and inhibits LPS-induced production of pro-inflammatory mediators, including NO, TNF-α, IL-1β, and IL-6 in a dose- and time-dependent manner. Mechanistically, these effects are supported by in vitro evidence of suppressed MAPK signaling pathway phosphorylation and inhibited NF-κB pathway activation. In vivo, in an LPS-induced endotoxin shock mouse model, Polyporusterone B alleviates multi-organ pathological damage, modulates tissue and serum pro-inflammatory cytokine levels, and improves hepatic/renal function evidenced by reduced serum AST, ALT, BUN, and CRE.

Notably, the inhibitory effects of Polyporusterone B on the MAPK and NF-κB pathways are currently supported exclusively by in vitro data, and in vivo validation of these pathway mechanisms remains pending. Despite this limitation, the consistent anti-inflammatory and organ-protective effects observed in both in vitro and in vivo systems suggest that Polyporusterone B holds potential as a therapeutic agent for inflammation-related diseases, with future studies focusing on in vivo pathway validation and efficacy in diverse inflammatory models to further advance its translational value.

## Data Availability

The datasets in the current study are included in the published article or available from the corresponding author on reasonable request.
